# Situated generosity in clinical care: A mixed-methods study of STI services in China

**DOI:** 10.1371/journal.pone.0352469

**Published:** 2026-06-26

**Authors:** Ke Zhou, Dorian Ho, Suzanne Day, Thomas Fitzpatrick, Katherine T. Li, Takhona Grace Hlatshwako, Danyang Luo, Zhuoheng Yin, Gifty Marley, Ligang Yang, Weiming Tang, C. Micha Belden, Joseph D. Tucker, Ruby Congjiang Wang

**Affiliations:** 1 University of North Carolina Project-China, Guangzhou, Guangdong Province, China; 2 Department of Health Policy and Management, University of North Carolina at Chapel Hill, Chapel Hill, North Carolina, United States of America; 3 Department of Medicine, University of North Carolina at Chapel Hill, Chapel Hill, North Carolina, United States of America; 4 Division of Allergy and Infectious Diseases, University of Washington, Seattle, Washington, United States of America; 5 Division of Infectious Diseases, School of Medicine, University of California, Los Angeles, Los Angeles, California, United States of America; 6 Behavioral, Social, and Health Education Sciences, Rollins School of Public Health, Emory University, Atlanta, Georgia, United States of America; 7 Department of Sexually Transmitted Disease Prevention and Control, Dermatology Hospital of Southern Medical University, Guangzhou, Guangdong Province, China; 8 Implementation Science Methods Unit, University of North Carolina at Chapel Hill, Chapel Hill, North Carolina, United States of America; 9 London School of Hygiene and Tropical Medicine, London, United Kingdom; Chinese Academy of Medical Sciences and Peking Union Medical College, CHINA

## Abstract

**Background:**

Generosity is a critical yet understudied dimension of prosocial behavior in healthcare. Existing research has focused on individual traits or professional values, with limited attention to how generosity is shaped in everyday clinical contexts. This study examines how healthcare professionals in China understand and enact generosity in sexually transmitted infection (STI) services, and how it is shaped by relational and organizational conditions.

**Methods:**

We conducted semi-structured interviews with 27 healthcare professionals and a focus group with four participants across five hospitals in Guangdong Province, China. Thematic analysis explored clinicians’ understandings and experiences of generosity. Crisp-set Qualitative Comparative Analysis (csQCA) examined configurations of relational and organizational conditions associated with reported generous practices. Data were analyzed using NVivo 12 and csQCA 4.0. Given uniform outcome presence, findings are interpreted as descriptive configurational patterns rather than causal effects.

**Results:**

Participants described generosity as discretionary practices beyond formal duties, including emotional support, flexible scheduling, and financial accommodations. These practices were associated with patient trust and collegial support, but also with emotional strain and boundary tensions in resource-constrained settings. Generosity was more often described in accounts involving clinicians’ engagement with patients’ broader social circumstances and experiences of supportive team environments. Overall, generosity appeared as a context-dependent practice shaped by interacting relational and organizational conditions.

**Conclusion:**

Generosity in STI care appears as a relationally embedded practice shaped by interactions among clinicians, patients, and organizational environments. It reflects the coupling of relational dynamics and institutional conditions that simultaneously enable and constrain discretionary care. Sustaining situated generosity therefore depends less on individual motivation than on organizational infrastructures that structure relational work in clinical settings.

## Introduction

### Conceptual background

Medicine functions as a sociocultural system in which norms, institutional logics, and relational expectations jointly shape clinical practice [1,2]. Compassion has long been recognized as a foundational affective orientation in care delivery [3], whereas recent scholarship has turned to generosity as its behavioral expression in clinical encounters [4].

In this study, generosity is conceptualized as a situated clinical practice involving the discretionary provision of emotional, temporal, or material resources, such as extended consultation time or financial flexibility, beyond formal role expectations and without immediate reciprocity [5]. Such practices are embedded in relational and organizational contexts.

Generosity is distinct from related constructs. Compassion denotes an affective response to suffering [6], while organizational citizenship behavior emphasizes extra-role functioning without accounting for how institutional and relational conditions shape practice in clinical settings [7]. Generosity is therefore examined as a situated practice enacted under relational and organizational constraint.

### Theoretical framing

This study integrates social exchange theory and virtue ethics to theorize generosity as a situated practice.

Social exchange theory explains discretionary clinical behavior through reciprocity, trust, and relational continuity [8–10]. Generosity emerges when reciprocal exchange structures create conditions of expected or perceived return. However, this account cannot explain its persistence under weak or absent reciprocity.

Virtue ethics locates generosity in professional moral orientation rather than exchange logic [11]. It explains sustained discretionary practice through moral commitment, professional identity, and normative commitments to care.

Generosity is therefore theorized as emerging through a dual-motivation mechanism: exchange structures generate opportunity conditions for discretionary action, while virtue-based moral commitment enables its continuation under weakened reciprocity. Situated generosity is thus a relational-moral practice produced through the coupling of exchange structures and moral dispositions.

### Research gaps and theoretical tensions

Prosocial behavior in healthcare is widely associated with improved patient experience and trust [10,12,13], yet its formation remains largely explained through isolated individual or organizational factors.

The relationship between generosity and clinician well-being is unresolved. While prosocial engagement may enhance meaning and professional identity [14], sustained discretionary effort under resource constraints may also produce burnout and emotional exhaustion [15–17]. Generosity thus operates simultaneously as relational value creation and structural strain response.

Existing approaches remain insufficient to explain how generosity is sustained under simultaneous relational demand and structural constraint. How generosity is configured across individual, relational, and organizational conditions therefore remains theoretically underdeveloped.

### Study context: STI services in China

STI services provide a particularly salient context for studying situated generosity. Patients often experience stigma, confidentiality concerns, and anticipated moral judgment [18–20], increasing reliance on relationally sensitive clinical practices.

In China, clinical practice is further shaped by Confucian relational ethics emphasizing *ren* (humanity), *guanxi* (relational embeddedness), and *mianzi* (reputation) [21,22], alongside systemic constraints of high patient volume, limited consultation time, and efficiency pressures [23–26]. This coexistence of moral relational expectations and structural constraint renders the setting analytically productive for examining how generosity is enacted under tension.

### Study aims and contribution

This study makes three main contributions. First, it offers a theoretical contribution by conceptualizing situated generosity as a configurational socio-moral practice produced through the interaction of relational and moral logics. Second, it provides a methodological contribution by demonstrating the analytical complementarity of thematic analysis and configurational comparison, integrating meaning-oriented interpretation with pattern-based cross-case analysis. Third, it offers an empirical contribution by generating insights from STI service settings in China, where moral relational expectations intersect with institutional constraints.

The study addresses three questions: how clinicians enact generosity, what consequences they associate with it, and which configurations of conditions sustain its routine enactment.

Overall, it conceptualizes generosity as a socially embedded and structurally conditioned practice rather than an individual trait or organizational behavior.

## Methods

### Study design and setting

This exploratory study adopts a qualitative-dominant configurational design integrating thematic analysis and crisp-set qualitative comparative analysis (csQCA) to examine patterns of generosity in clinical care. Thematic analysis explores how generosity is understood and enacted in everyday practice, while configurational comparison identifies recurring patterns of contextual conditions across cases.

Routine generosity was observed across all cases. Accordingly, the configurational analysis focuses on patterns of co-occurring conditions rather than contrasts between presence and absence of the outcome.

The study was conducted among sexual health professionals in Guangdong Province, China, building on prior research on pay-it-forward interventions in similar settings [27]. In this context, generosity (*kangkai-dafang*) encompasses both material and emotional support in clinical interactions.

### Ethical statement

Ethical approval was obtained from the Dermatology Hospital of Southern Medical University (approval no. IIT-2023-134) and the University of North Carolina at Chapel Hill (approval no. 21-1667). All participants provided written and oral informed consent. Participation was voluntary and confidential, with a small compensation of 50 RMB (~7 USD). Further details on ethical, cultural, and inclusivity considerations are provided in the Supporting Information (S1 File).

### Participants and data collection

Between 1 May and 28 June 2024, we conducted 27 semi-structured interviews with sexual health professionals in Guangdong Province. Participants were recruited via a gatekeeper approach; 27 of 35 eligible individuals agreed to participate (response rate 77.0%). Purposive sampling ensured inclusion across clinical roles and seniority levels. Participants had an average of 16 years of clinical experience (range 2-35 years; Table 1).

**Table 1:** Sociodemographic and Professional Characteristics of Participants (N = 27).

Characteristic	Category	n (%)
**Age (years)**	< 30	4 (14.8)
	30-39	13 (48.1)
	40-49	7 (25.9)
	≥ 50	3 (11.1)
**Gender**	Male	5 (18.5)
	Female	22 (81.5)
**Highest Educational Attainment**	College/Undergraduate	10 (37.0)
	Master’s degree	14 (51.9)
	Doctoral degree	3 (11.1)
**Clinical Experience (years)**	0-5	9 (33.3)
	6-10	11 (40.7)
	11-20	3 (11.1)
	> 20	2 (7.4)
**Professional Role**	Physician	9 (33.3)
	Nurse	8 (29.6)
	Medical Student	8 (29.6)
	Laboratory Technologist	2 (7.4)

Interviews were conducted in Mandarin by two native Chinese-speaking researchers using a pilot-tested guide (S2 File), lasting 45-75 minutes. Thematic saturation was reached after 24 interviews.

To support member checking, a two-hour focus group with four original participants was held on 26 June 2024. Preliminary findings were presented to validate identified themes and refine relationships between generosity and routine practice.

All interviews and the focus group were audio-recorded, transcribed verbatim, and anonymized. Transcripts were translated into English by bilingual researchers. To ensure conceptual equivalence across languages, translations were reviewed through an iterative bilingual consensus process involving systematic comparison of Mandarin transcripts and English translations, with discrepancies resolved through discussion among the research team. Although formal back-translation was not conducted, this process served as a functional equivalent to enhance semantic and conceptual fidelity.

The high proportion of female participants (81.5%) reflects the gender composition of STI service settings in China, although it may influence how generosity is articulated and experienced. The inclusion of medical students provided insight into a transitional stage of professional socialization, complementing the perspectives of more experienced clinicians. Recruitment through gatekeepers facilitated access to clinical settings but may have introduced selection bias toward participants more willing to reflect on or discuss generous clinical practices..

### Thematic analysis

Qualitative data were analyzed using a six-phase inductive thematic analysis approach [28]. Two researchers with backgrounds in sociology and medicine independently coded the dataset in NVivo 12 using a structured codebook (S3 File). Coding was refined through iterative consensus, with discrepancies resolved through discussion of original transcripts to ensure interpretive consistency [29].

Rather than treating themes as isolated categories, analysis focused on how generosity is embedded in routine clinical interactions.

### Configurational analysis of contextual conditions

A set-theoretic configurational comparison examined patterns of co-occurring conditions across cases [30–32]. The dataset included 27 cases and four conditions derived from the qualitative analysis.

#### Calibration of conditions.

Four conditions were calibrated into binary crisp sets (0 = absence, 1 = presence) based on theoretically and empirically informed anchors (Table 2), following set-theoretic calibration principles [31,33]. A detailed stepwise overview of the analytical procedure is provided in the Supporting Information (S2 Table) for transparency.

**Table 2:** Calibration Anchors and Decision Rules for csQCA Conditions.

Condition / Factor	Concept Definition	Calibration for 0 (Absence)	Calibration for 1 (Presence)
**Outcome: Routine Practice of Generosity**	Whether the practitioner actively incorporates generous behaviors into daily clinical work.	Exclusion Rule: Practitioner describes adhering strictly to minimum standard care, or explicitly states they do not perform extra-role behaviors. *(e.g., “I just follow the protocol; I don’t have time for extras.”)*	Inclusion Rule: Practitioner provides concrete examples of extra-role behaviors, such as devoting extra time for health education, maintaining contact outside office hours, or waiving fees for low-income patients. *(e.g., “Even if they are difficult, I still try to help.”)*
**C1: Attitudes towards Social Complexity**	Willingness to maintain generosity towards patients with complex social backgrounds or stigmatized conditions.	Exclusion Rule: Narrative explicitly states withholding generosity from “difficult” or “arrogant” patients, or expressing a desire to “give up” on them.	Inclusion Rule: Narrative expresses empathy specifically for the patient’s social struggle or describes maintaining high-quality care despite the patient’s stigma or difficult behavior.
**C2: Team Support / Modeling**	Perception of the collegial environment and exposure to generous role models.	Exclusion Rule: Participant mentions working in silos, lack of recognition from leaders, or describes team relations as purely transactional interactions.	Inclusion Rule: Participant explicitly mentions “unity,” “family-like atmosphere,” or cites specific examples of help, mentorship, or generous practices modeled by colleagues.
**C3: Emotional Exhaustion**	State of emotional and physical exhaustion related to work.	Exclusion Rule: No indication of emotional exhaustion; fatigue framed as temporary or purely physical.	Inclusion Rule: Explicit indication of emotional exhaustion, depersonalization, or reduced professional efficacy.
**C4: Attitudes towards Patient Compliance**	Perception of patient interaction and its influence on care provision.	Exclusion Rule: Narrative links generosity to patient obedience, cooperation, or “good behavior.”	Inclusion Rule: Narrative indicates generosity is provided regardless of patient behavior or compliance.

Conditions were selected based on theoretical relevance and salience in the qualitative dataset [34]. For example, experiences of emotional exhaustion were coded as present when participants described emotional depletion, detachment from patients, or reduced professional efficacy.

Two researchers independently applied calibration rules, with discrepancies resolved through discussion (S3 File). The resulting dichotomized dataset is presented in S3 Table.

### Truth table and analysis

A truth table summarized observed combinations across cases (Table 3). Configurational pattern summaries were generated using csQCA software (v4.0) [35], with a consistency threshold of 0.75 [32].

**Table 3:** Truth Table of Condition Combinations Across Cases.

Social complexity	Patient compliance	Emotional exhaustion	Team support	Number of cases	Outcome (routine generosity)	Raw consistency
1	0	1	1	7	1	1
1	0	0	1	6	1	1
1	0	1	0	3	1	1
1	1	1	1	3	1	1
1	1	0	1	2	1	1
0	0	1	1	2	1	1
0	1	0	0	1	1	1
1	1	1	0	1	1	1
0	0	0	1	1	1	1
0	1	1	1	1	1	1

Note: Because routine generosity was present in all cases, the truth table is presented as a descriptive summary of observed configurations and should not be interpreted as evidence of causal necessity or sufficiency.

Given the study’s focus on routine clinical generosity, csQCA is used as a configurational tool to organize and compare patterns across cases rather than to make causal claims about conditions producing the outcome.

The crisp-set calibration reduces constructs into binary categories based on theoretically informed coding rules. This approach facilitates cross-case comparison while acknowledging the loss of within-case nuance.

### Interpretation

Results are interpreted as descriptive configurational profiles of co-occurring conditions. Configurational summaries are reported to support transparency.

## Results

### Thematic analysis results

#### Conceptualizing and Enacting Generosity.

Participants conceptualized generosity as rooted in altruism, care, and moral obligation, while also distinguishing between conceptual understanding and practical enactment. Rather than an individual disposition, generosity was described as a situated relational practice shaped by professional values, patient needs, and social expectations within clinical settings (Table 4). This suggests generosity is best understood as a practice through which moral commitments are translated into clinical action. As one doctor explained, “My behaviors come from sympathy and concern for patients,” while another emphasized that “the generosity of the entire team promotes a better workplace for everyone,” highlighting its embeddedness within collective clinical norms.

**Table 4:** Definitions and Enactments of Generosity in Clinical Settings.

Conceptual understandings of generosity	Illustrative enactments in practice
Altruism, care, and moral obligation	Psychological support and verbal reassurance
Professional values and expectations	Additional consultation time for complex patients
Trust and rapport in clinician-patient relationships	Fee reduction or financial accommodation
Influence of social roles and workplace norms	Sharing personal contact information (e.g., phone/WeChat)

Participants consistently distinguished between routine professional duties and discretionary practices that exceeded standard care. Basic empathy, respectful communication, and adherence to clinical protocols were generally regarded as professional obligations. By contrast, spending additional time with complex patients, providing financial accommodation, arranging additional follow-up, or remaining accessible beyond formal hours were more often described as expressions of generosity. One clinician noted this distinction explicitly, stating that routine diagnosis and treatment are professional responsibilities, whereas “spending extra time to explain things repeatedly or arranging additional follow-up reflects a personal decision to provide more support.”

Emotional support was the most frequently reported form of generous engagement (25/27, 92.6%). While emotional support may be considered part of routine clinical care, participants often referred to forms of psychological support that exceeded standard expectations, including extended counseling, repeated explanations, anticipatory guidance, and ongoing support for patients facing stigma, uncertainty, or complex social circumstances. All clinicians described allocating additional consultation time for complex cases, 81.5% reported financial accommodations, and 29.6% facilitated continuity of care by sharing personal contact information such as WeChat for post-consultation access.

### Relational and professional consequences of generous practice.

Participants described consequences of generosity across relational outcomes in patient interactions and professional outcomes related to clinicians’ experiences and well-being (Table 5).

**Table 5:** Relational and Professional Consequences of Generous Practice.

Relational consequences
**For patients:** Reduced anxiety and psychological distress Improved engagement and cooperation **For clinicians:** Strengthened clinician-patient relationships Increased patient trust and reciprocity
**Professional consequences**
**For clinicians:** Professional fulfillment and moral satisfaction Skill development and learning opportunities Emotional exhaustion and fatigue Increased workload and time pressure **For services:** Improved working environment Resource strain

#### Relational consequences.

Generous practices were associated with stronger therapeutic relationships, reduced patient anxiety, and improved treatment adherence. As one senior doctor observed, “Generosity helps improve the effect of comprehensive treatment,” while a junior clinician noted that “psychological support can reduce anxiety and improve quality of life.” In STI care, where stigma often disrupts engagement, these effects were considered particularly important for continuity of care.

Generosity also reinforced reciprocal trust within clinician-patient relationships. When patients expressed gratitude or acknowledged additional efforts, clinicians often felt encouraged to continue investing time and support. As one participant described, “Some patients add me on WeChat after consultation and send updates; when they thank me or report improvement, the extra effort feels worthwhile,” illustrating a relational feedback loop that sustains generous practice.

#### Professional consequences.

For clinicians, generosity was associated with professional fulfillment, moral purpose, and skill development. Junior doctors and students emphasized that engaging with complex cases provided learning opportunities beyond standard protocols and strengthened clinical expertise. Many also described moral satisfaction from supporting patients facing stigma or vulnerability.

However, sustaining generosity required substantial emotional and temporal investment, producing a recurring tension between fulfillment and emotional exhaustion. Three mechanisms of strain were identified.

**Emotional labor.** Responding to patients’ emotional demands was often described as exhausting. When expectations could not be fully met, disappointment or criticism gradually reduced clinicians’ emotional capacity to sustain support.

**Boundary erosion.** Practices such as sharing personal contact information or responding outside working hours blurred professional boundaries. Some clinicians reported increasing patient expectations for continuous availability, placing additional demands on limited time and emotional resources.

**Structural pressures.** High patient volume and limited consultation time constrained the sustainability of generous practices. As one physician noted, “your generosity to a patient may disrespect your colleague’s time,” highlighting how discretionary care can generate systemic pressure within teams.

As one clinician summarized, generosity was often sustained despite personal costs, driven by responsibility toward patients who might otherwise receive inadequate care, including efforts to extend consultations or work beyond formal hours when necessary.

### Conditions associated with generous practice

Participants identified facilitators of generous practice across patient-, provider-, and system-level contexts. These conditions were described as dynamically interacting rather than operating independently, shaping how generosity is enacted in routine clinical work.

At the patient level, strong rapport, perceived vulnerability, and expressions of gratitude reinforced clinicians’ willingness to provide additional support. Social stigma in STI care also shaped practice, prompting more protective and nonjudgmental approaches to maintain engagement.

At the provider level, team culture and role modeling were key influences. Observing senior clinicians’ behaviors helped normalize generous practice, with participants noting that such practices were learned through daily exposure rather than formal instruction.

At the system level, organizational support was consistently identified as essential. Adequate staffing, administrative flexibility, and institutional backing enabled clinicians to allocate time beyond standard consultation structures. Without such support, discretionary practices were difficult to sustain, as one clinician noted: “If there is no support from higher-level administration, it is very difficult for us to continue these kinds of generosity-related initiatives for patients.”

Taken together, these conditions form the empirical basis for the subsequent configurational analysis, which examines how they co-occur across cases.

### Configurational analysis of contextual conditions

We analyzed data from 27 participants and calibrated four conditions (S4 Table) according to pre-specified criteria (S1 Table). The conditions were derived from qualitative findings to ensure conceptual distinctiveness across domains. Routine generosity was uniformly present across all cases. The truth table (Table 3) therefore focuses on observed configurations of contextual conditions.

Table 6 shows the distribution of conditions across cases. SOCIAL COMPLEXITY (0.81) and TEAM SUPPORT (0.81) were most prevalent, followed by EMOTIONAL EXHAUSTION (0.63) and PATIENT COMPLIANCE (0.30). This indicates that generosity is observed across heterogeneous clinical contexts rather than being restricted to a specific environment. Following standard QCA notation, the presence of a condition is indicated by uppercase letters (e.g., SOCIAL COMPLEXITY), while absence is denoted by a tilde preceding lowercase letters (e.g., ~ social complexity) [36].

**Table 6:** Distribution of Conditions Across Cases.

Condition label	Present Cases (n)	Sample proportion
**SOCIAL COMPLEXITY**	22	0.81
**TEAM SUPPORT**	22	0.81
**EMOTIONAL EXHAUSTION**	17	0.63
**PATIENT COMPLIANCE**	8	0.30

Note: Number of cases indicates the frequency with which each condition was present (coded as 1) across the sample (n = 27). Proportions were calculated accordingly. Frequencies indicate the prevalence of each condition across cases and should be interpreted descriptively rather than as evidence of causal importance.

### Configurational patterns

Table 7 presents recurring configurations of conditions in cases reporting generosity. Each configuration represents a recurring combination of conditions identified in contexts where generosity was reported (denoted × , e.g., SOCIAL COMPLEXITY × TEAM SUPPORT) [36].

**Table 7:** Combinations of Conditions Observed in Contexts Reporting Generosity.

Combination of conditions	Present Cases (n)	Sample proportion
**~patient compliance × TEAM SUPPORT**	15	0.56
**SOCIAL COMPLEXITY × EMOTIONAL EXHAUSTION**	14	0.52
**SOCIAL COMPLEXITY × TEAM SUPPORT**	18	0.67
**EMOTIONAL EXHAUSTION × TEAM SUPPORT**	13	0.48
**~social complexity × PATIENT COMPLIANCE × ~emotional exhaustion × ~team support**	1	0.04

Note: The combinations reported in this table represent recurring patterns of co-occurring conditions observed in cases reporting generosity. They should be interpreted as descriptive contextual patterns rather than causal configurations or solution pathways.

The most frequent patterns were SOCIAL COMPLEXITY × TEAM SUPPORT (0.67), ~ patient compliance × TEAM SUPPORT (0.56), SOCIAL COMPLEXITY × EMOTIONAL EXHAUSTION (0.52), and EMOTIONAL EXHAUSTION × TEAM SUPPORT (0.48). One rare configuration (~social complexity × PATIENT COMPLIANCE × ~emotional exhaustion × ~team support) was observed in a single case.

These configurations indicate that generous practice is associated with multiple interacting relational and organizational conditions rather than a single dominant pattern.

### Recurring contextual patterns across cases

Four recurring configurational patterns were identified.

**Supported patient-centered practice (SOCIAL COMPLEXITY × TEAM SUPPORT).**

This pattern reflects contexts in which clinicians respond to vulnerable patient needs within supportive team environments, where organizational culture reinforces discretionary care.

**Team-supported responses to challenging patients (~patient compliance × TEAM SUPPORT).**

This captures situations in which clinicians maintain generous practices despite perceived patient non-compliance, supported by collegial environments that buffer relational strain.

**Generosity under strain (SOCIAL COMPLEXITY × EMOTIONAL EXHAUSTION).**

This pattern reflects cases in which clinicians report emotional exhaustion while continuing to respond to highly vulnerable patients, indicating sustained discretionary effort under pressure.

**Co-occurrence of emotional exhaustion and team support (EMOTIONAL EXHAUSTION × TEAM SUPPORT).**

This illustrates that emotional exhaustion and collegial support can coexist, with team structures partially mitigating but not eliminating workload-related strain.

### Summary

Across configurations, generosity emerges in diverse combinations of relational, professional, and organizational conditions. Rather than a single dominant pathway, the findings highlight heterogeneity in the contexts in which generosity is enacted.

These results complement the thematic analysis by providing a structured cross-case representation of how contextual conditions co-occur in clinical practice.

## Discussion

This study conceptualizes generosity in clinical care as a situated socio-moral practice produced through the interaction of relational expectations, organizational constraints, and clinician agency. Rather than an individual disposition or stable organizational output, generosity emerges as a contextually produced practice embedded in relational and institutional configurations.

### Situated generosity as a relational-moral practice

By integrating social exchange theory [8] and virtue ethics [37], this study shows that generosity is neither fully explained by reciprocity-based exchange nor by individual moral disposition alone. Instead, exchange structures generate the relational conditions under which discretionary care becomes possible, while virtue-based moral commitments sustain such practices when reciprocity is weak or absent. This dual mechanism reframes generosity as an emergent practice sustained through asymmetric exchange conditions and moral commitment under relational uncertainty.

These findings extend existing accounts of organizational citizenship behavior [7] by demonstrating that extra-role clinical practices are not merely responses to organizational norms but are embedded in relational asymmetries, stigma, and patient vulnerability. In such contexts, reciprocity is partial, delayed, or absent, requiring clinicians to rely on moral commitments rather than exchange balance to sustain care practices.

### Generosity as tensioned practice

The findings further indicate that generosity is not reducible to either positive engagement or emotional exhaustion, but instead emerges through their con-presence. This is consistent with research on compassion fatigue [38,39] and emotional labor [40], which similarly highlights the co-existence of meaningful engagement and emotional burden in clinical work. Emotional labor, workload pressure, and boundary negotiation are not simply external constraints but constitutive conditions of discretionary care, producing a persistent tension in which care and strain are co-generated.

**Fig 1:**
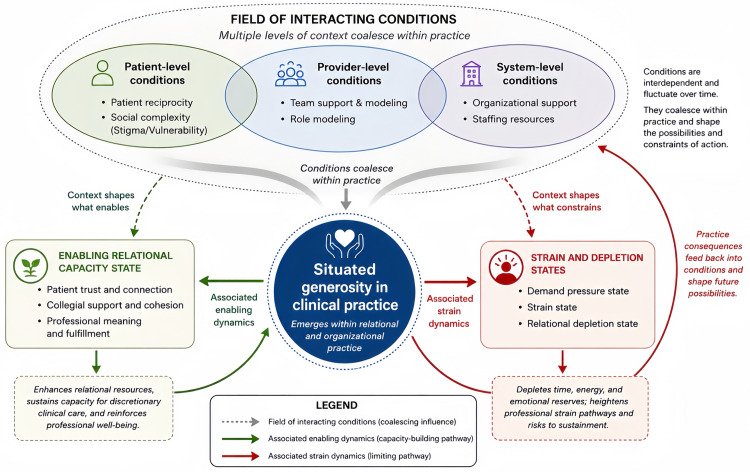
Conceptual model of situated generosity in clinical practice. The model integrates findings from thematic and cross-case analyses to illustrate how patient-, provider-, and system-level contexts shape situated generosity through enabling and limiting processes. It represents a conceptual synthesis of recurring patterns observed across cases rather than a depiction of causal relationships.

Fig 1 presents a conceptual synthesis of the findings, illustrating how patient-, provider-, and system-level contexts jointly shape situated generosity through interacting enabling and limiting processes.

### Configurational production of generosity

Across cases, generosity appears to emerge from configurations of relational trust [41,42], collegial support [43] patient vulnerability [44,45], and organizational pressure rather than from any single determinant. These patterns challenge variable-centered explanations of prosocial behavior by showing that generosity is structurally conjunctural: it arises from combinations of reinforcing and constraining conditions rather than isolated factors.

In particular, stigma and patient vulnerability can function paradoxically as both constraints and motivators of discretionary care [46], depending on their interaction with organizational workload and team support. This reinforces the importance of treating generosity as an emergent property of contextual configurations rather than a predictable outcome of individual attitudes or institutional resources.

### Digital communication and boundary reconfiguration

Digital communication practices, particularly informal platforms such as WeChat, further illustrate how boundaries of care are reconfigured in resource-constrained settings [47]. Rather than functioning as a neutral tool, digital communication extends relational continuity beyond formal clinical encounters while simultaneously intensifying temporal and emotional demands on clinicians.

This dual effect highlights how digital infrastructures reshape the temporal and spatial boundaries of clinical work. In contexts of limited formal follow-up systems, informal digital engagement becomes both an adaptive mechanism for sustaining care and a source of intensified professional exposure.

### Implications

These findings suggest that sustaining generosity in clinical care depends less on individual motivation than on the relational and organizational infrastructures in which care is embedded. Supporting discretionary care therefore requires attention to workload distribution, team-based support, and institutional recognition of emotional labor.

At the same time, the findings caution against unstructured reliance on informal digital communication as a substitute for formal care systems. Without institutional guidance, such practices may shift care burdens onto clinicians and intensify boundary ambiguity.

### Methodological considerations and limitations

This study should be interpreted in light of several methodological considerations that shape the scope and inference of the findings.

First, the use of csQCA in a context where routine generosity was present across all cases limits the analytical capacity to assess necessary or sufficient conditions in a traditional set-theoretic sense [48]. The absence of negative cases precludes systematic comparison between the presence and absence of the outcome, and therefore the configurational results should be understood as descriptive patterns of co-occurring conditions rather than causal explanations [49].

Second, while the integration of thematic analysis and configurational comparison allows for both interpretive depth and cross-case structuring, the study remains dependent on a relatively small, purposively sampled dataset. Recruitment through gatekeepers may have introduced selection bias toward clinicians more willing to reflect on or articulate discretionary practices.

Third, although analytic rigor was supported through peer debriefing and iterative team discussion, the interpretive process inevitably reflects the disciplinary positioning of the research team, particularly in coding and calibration decisions.

Finally, the transferability of findings is constrained by the specific institutional and social context of public STI clinics in one region of China, where stigma, workload pressure, and resource constraints may intensify both the visibility and fragility of discretionary clinical practices.

Taken together, these considerations suggest that the study’s contribution lies not in causal inference, but in providing a configurationally informed, context-sensitive account of how generosity is enacted under structurally constrained clinical conditions.

## Conclusion

This study advances a configurational understanding of situated generosity as a relational-moral practice embedded within institutional structures of constraint and interdependence. The findings show that discretionary care emerges through interactions among clinicians, patients, and healthcare systems characterized by asymmetry, stigma, and resource pressure. Rather than functioning as a supplementary behavior, generosity forms part of the relational infrastructure through which clinical care is sustained under structural limitation.

From a systems perspective, this implies that clinical care cannot be fully understood without accounting for the relational infrastructures through which it is enacted. However, such infrastructures also redistribute institutional burdens onto clinicians’ emotional and relational labor, raising questions about the sustainability of discretionary care under intensifying workload and digital connectivity.

Future research should examine how different institutional configurations shape the emergence, persistence, or erosion of discretionary care, particularly through comparative studies across clinical specialties, longitudinal designs, and contexts where such practices are absent.

## Supporting information

S1 Table Key QCA terms and definitions used in the study. (PDF)

S2 Table Eleven-step protocol for crisp-set QCA analysis of generosity. (PDF)

S3 Table Dichotomized dataset for csQCA conditions and outcome. (PDF)

S4 Table Bivariate correlations among csQCA conditions. (PDF)

S1 File Inclusivity in global research questionnaire. (PDF)

S2 File Semi-structured interview guide used in the study. (PDF)

S3 File Thematic coding framework and csQCA calibration rules. (PDF)
