# Supplementary material for: Situated generosity in clinical care: A mixed-methods study of STI services in China
Source: PLoS One. 2026 Jun 26;21(6):e0352469. doi: 10.1371/journal.pone.0352469 (PMC13308865; doi:10.1371/journal.pone.0352469)
Supplement: S1 Table — (PDF) [file pone.0352469.s001.pdf]

S1 Table. Key QCA terms and definitions used in the study.

Table 1. Key terms in qualitative comparative analysis (QCA)

Key term	Definition
Complex solution	No assumptions are made about the logical remainders in this solution. A subset of the two other solution terms (intermediate and parsimonious)
Consistency	How well a path and the full solution reflect the data
Configuration	Combination of risk and protective conditions (present and absent) in the case
Condition	Factor that contributes to explanation of the outcome
Coverage	Percentage of the paths and solutions explained by the cases
Crisp set QCA	This form of QCA allows only binary forms of conditions. In set theory terms, conditions fall in (labelled as 1) or out (labelled as 0) of the sets
Equifinality	Allows for equal but different condition combinations to an outcome
Intermediate solution	Uses a combination of both theory and the empirical cases to determine the paths. The empirical paths will never be contradicted in this solution
Limited diversity	There are more paths possible than empirical cases. This is shown by the number of logical remainders
Logical AND (*)	Intersection of sets
Logical remainder	Possible paths for which there are no empirical examples from the cohort
Logical minimization	Process through which configurations are minimized. If two cases lead to the same outcome and are the same except for one variable, the variable that differs is not causal to the outcome and is eliminated (for more information, see section 2 in the supplemental data online)
Necessity	A condition is necessary if it is always present when the outcome is present
Parsimonious solution	The most simple solution that uses mainly theory with the empirical cases to derive the path solutions
Path	Combination of conditions that are sufficient to result in an outcome. There may be one or many cases in a path. Conditions are combined with the logical AND
Raw coverage	Percentage of total cases covered by a path (number of cases divided by the total number of cases)
Set	Series of objects, ideas or states of being that are well defined
Solution	All the minimized paths that result from the analysis. There are three types of solution: parsimonious, intermediate and complex
Sufficiency	A condition is sufficient when combined with one or more conditions; it is causal for the outcome
Truth table	All the condition combinations empirically found
Unique coverage	Percentage of the total cases covered only in this path (number of unique cases divided by the total number of cases)

Adapted from Short et al. (2020).
