# Supplementary material for: Situated generosity in clinical care: A mixed-methods study of STI services in China
Source: PLoS One. 2026 Jun 26;21(6):e0352469. doi: 10.1371/journal.pone.0352469 (PMC13308865; doi:10.1371/journal.pone.0352469)
Supplement: S2 Table — (PDF) [file pone.0352469.s002.pdf]

S2 Table. Eleven-step protocol for crisp-set QCA analysis of generosity.

Step	Action	Explanation and application
**1**	Determine guiding theory	Review of literature on generosity, especially in healthcare settings, to inform theory
**2**	Determine the outcome(s)	Presence and absence of one outcome of choice (generosity, cynicism); operationalized based on existing theory of what constitutes generosity or cynicism in clinical encounters
**3**	Determine the cases in which to explore the findings	Cases should span a range of demographics and professional characteristics. For ideal analysis, one group of cases should demonstrate generosity, while all other cases should demonstrate cynicism
**4a**	Determine the key factors to test for the outcomes and data collected	Key factors (predictors) will be determined based on prevalent themes inductively derived from semi-structured interviews; examples of factors that might be relevant include salary, perceived career advancement, perceived job security, sufficiency of staff and equipment, etc.
**4b**	Formation of the conditions, set membership, and calibration	Factors will be dichotomized at reasonable cut points based on theory and discussion; some factors may be clear-cut, whereas others may need deliberation
**5**	Create a truth table	Map all values (0 or 1) for each factor and outcome for each included case in a matrix
**6**	Examine the truth table and resolve any conflicts	Ideally, a truth table will demonstrate high consistency, where similar combinations of factors result in the same outcome. Consistency >0.75 is optimal, whereas consistency below this score requires iterative refinement of cut points or addition of new factors
**7**	Conduct the necessity analysis	Necessity analysis identifies the most prevalent factors for a particular outcome; necessity is based on consistency and coverage, or the percentage of cases

Step	Action	Explanation and application
		covered by a particular factor; consistency >0.9 and coverage >0.5 are a priori cut points to determine necessity
**8**	Conduct the sufficiency analysis and generate path solutions	Sufficiency analysis identifies the combinations of factors sufficient to induce a particular outcome; sufficiency is based on consistency and coverage, with a priori cut points at consistency >0.75 and coverage >0.5 Sufficiency analysis results in 'path solutions' comprising of these factor combinations; these solutions can be logically minimized with Boolean logic (software) to simpler forms; complex (least simplified), intermediate, or parsimonious (most simplified) solutions can be reported, based on what balance of case orientation and established theory is desired
**9**	Evaluate the parameters of fit	Evaluate consistency and coverage for all paths (solutions), which indicates whether the QCA models fit the empirical data observed
**10**	Determine the symmetrical influence	Determine whether the factors present in paths leading to one outcome are those that are absent in paths leading to its converse; causal asymmetry is assumed (i.e. one set of paths leading to one outcome, a different set of paths [combinations of factors] explaining another outcome)
**11**	Interpret the results	Interpret the paths identified considering existing theory or generate new theory explaining results

Adapted from Short et al. (2020), with methodological insights from Rihoux and Ragin (2009), Schneider and Wagemann (2012).
