# Supplementary material for: Situated generosity in clinical care: A mixed-methods study of STI services in China
Source: PLoS One. 2026 Jun 26;21(6):e0352469. doi: 10.1371/journal.pone.0352469 (PMC13308865; doi:10.1371/journal.pone.0352469)
Supplement: S3 Table — (PDF) [file pone.0352469.s003.pdf]

S3 Table. Dichotomized dataset for csQCA conditions and outcome.

Case Number	SOC CMPX	PT COMP	EM EXH	TEAM SUP	Outcome
1	1	0	1	0	1
2	1	0	1	0	1
3	1	1	1	1	1
4	1	0	0	1	1
5	1	0	1	1	1
6	1	1	1	0	1
7	0	0	1	1	1
8	1	0	1	1	1
9	1	0	1	1	1
10	0	0	0	1	1
11	1	0	0	1	1
12	1	0	1	1	1
13	0	0	1	1	1
14	1	0	0	1	1
15	1	1	1	1	1
16	1	0	0	1	1
17	1	0	1	0	1
18	0	1	1	1	1
19	1	0	0	1	1
20	1	0	1	1	1
21	1	0	1	1	1
22	0	1	0	0	1
23	1	1	1	1	1
24	1	1	0	1	1
25	1	0	0	1	1
26	1	0	1	1	1
27	1	1	0	1	1
