# Supplementary material for: Situated generosity in clinical care: A mixed-methods study of STI services in China
Source: PLoS One. 2026 Jun 26;21(6):e0352469. doi: 10.1371/journal.pone.0352469 (PMC13308865; doi:10.1371/journal.pone.0352469)
Supplement: S4 Table — (PDF) [file pone.0352469.s004.pdf]

S4 Table. Bivariate correlations among csQCA conditions.

	SOC CMPX	*PT COMP*	*EM EXH*	*TEAM SUP*
**SOC CMPX**	1			
**PT COMP**	-0.10827	1		
**EM EXH**	0.029251	-0.00622	1	
**TEAM SUP**	0.018182	-0.10827	-0.16819	1
