# Supplementary material for: Situated generosity in clinical care: A mixed-methods study of STI services in China
Source: PLoS One. 2026 Jun 26;21(6):e0352469. doi: 10.1371/journal.pone.0352469 (PMC13308865; doi:10.1371/journal.pone.0352469)
Supplement: S2 File — (PDF) [file pone.0352469.s006.pdf]

**S2 File. Semi-structured interview guide used in the study.**

**A. English version of interview guide.**

Semi-structured interviews with open ended questions that flow according to participant response.

A. Basic Information

1.Can you share relevant personal details? e.g.

a) Gender

b) years of experience

c) your current position and organisation, and why chose this? (reputation of training institution, private/public hospital)

d) for doctors: how much is your registration fee?

e) for both doctors and nurses: how many patients do you need to see per day? Do you see more STD patients or dermatology patients?

2.What are your previous jobs/career track/medical education?

3.Any other relevant details for supplement?

B. Definition and Experience of Generosity

1.What comes to mind with the word "generosity"? Do these words below strike you as clinical generosity?

index card for participant to choose: goodwill, conscience, good doctor, empathy, human care, moral obligation, ethics, humanitarianism, social responsibility, altruism, reputation, religious (Fu Bao), doing a good deed every day, good people will get reward, etc.

2.Would you take extra time, or give your own money, to help patients in need?

3.Do you feel that you are able to act this way in your clinical encounters? If so, how frequently do you act this way? Can you tell us about one situation that comes to mind? e.g. extraordinary vs some, episodic vs persistent, or it depends/never done that.

4.What impacts, positive or negative, could generosity have on patient care? On the operation of the healthcare system?

Tips: explore the reason behind their decision. -slows down workflow, cannot fix everyone's problems, etc.

5.Are there health professionals that you know who always seem generous, and how did that impact the team? What do you think and how do you feel when receive generosity from your colleagues?

If not, is there an icon or historical figure you can reference when considering a "generous clinician"?

Tips: For individual-social norms and peer effects (feeling a certain pressure or expectation and then imitating that behavior); for team-organizational cohesion and effectiveness are enhanced; A good team atmosphere; for the system-a more cooperative, caring, and fair medical setting.

C. Factors of Generosity

Next, we will discuss the potential factors that affect generosity of clinicians' behaviors or communication. The factors below are among individuals, patients, and clinical system. For the clinical encounter aspect, it may include patient social factors, patient behavior, physician-patient rapport; for the clinicians as individuals, it may include moral obligation, professional pride and satisfaction, clinician burnout; and for system level, it may include team collaboration and support, system resources, salary and other factors.

1.We will now explore clinical encounters. How might these factors affect your generosity?
Clinician Manner:
How do you respond verbally/physically when a patient describes his circumstance (e.g. a verbal reassurance, a nod of understanding, a follow-up)? Why or why not?
What's your attitude and response towards the patients whose needs or requests might go beyond the normal scope of the consultation?
Patient Reciprocity: How does a patient respond to or reciprocate to your generosity? Verbal thanks or an action as feedback?
Social complexity: What patient factors make you more or less generous? their disease, their social complexity? demographic, work and life circumstances. E.g. age, women, MSM, sex workers, etc.
Stigma: Could you explain more about their social complexity, for example how stigma and social discrimination of sexual health compared to dermatology, that might affect patient behavior, and how that affects your provision of generosity as a clinician?

2.Let's talk about clinician factors. How might these factors affect your generosity?
Accountability: Do you feel personally accountable for patient outcomes?
Empathy: Do you feel personally concerned for the well-being of all your patients?
What enables you to build better rapport with the patient more easily?
Work: How do you feel about your job? What do you like about it?
What do you not like, or wish could improve or change (both realistic and aspirational)? Do those factors make you less generous in your job?
Burnout/depersonalization: How often do you feel emotionally fatigued or withdrawn? (never, once a month, once week, more than once a week)
What might make you more burned out - greater number of patient workload, complexity of the patient presentation etc?
How would this impact your level of generosity towards patients or colleagues?

3.We will now move onto systemic factors. How might these factors affect your generosity?
-[index card for participant to choose the most affective factors]
Team size – Number of health professionals involved in patient care
Whether staffing is sufficient to meet patient needs
Resources – whether equipment and technology are sufficient to meet patient needs
Ownership – public or private hospital
Compensation – salary above/below (provincial?) averages? If possible, could you tell us your annual salary?

4.What might dis/incentivive you to choose or stay at an organization?
For younger: are you used to this job by now?
For those more experienced: research task/office politics/bureaucracy/extra paperwork regarding non-clinical issues
-[index card for participant to choose the most affective factors]
-Adequate payment and compensation (salary and bonus)
-Professional development opportunities (education, training, career prospects)
-Job security (temporary posts versus permanent roles/ "Bianzhi" or iron rice bowl)
-Working environment (workload, staffing and support)
-Working conditions and resources (essential equipment and high-quality facilities)
-Office politics/bureaucracy/extra paperwork regarding non-clinical issues
-Location (convenient transportation)
-Reputation of provider (brand and ranking)}

5.Which three of those factors are most important in influencing how generously you act towards a patient? [index card with the above factors included for participant to choose]
Encounter-level
Clinician-level
System-level

6.Are there other factors that you believe may influence generosity in the clinical encounter?

7.How could we create a work setting with anti-cynicism interventions in order to promote generosity in medicine and in turn, reduce adverse outcomes?

8. How can generosity be sustained and passed on?

Conclusion: Do you have any other questions or related suggestions?

Mandarin version of interview guide

B. **Mandarin version of interview guide.**

一、基本信息
1.我们首先需要了解您的个人基本信息。
性别
工作年限
所在单位和职位（单位名声、级别，公立\私人医院）；以及您为何选择了这里？
对医生：您的挂号费是多少？
对医生和护士等：您一天大概看多少个病人？平时看性病患者比较多，还是皮肤病患者比较多？
2.请介绍一下您的先前的教育经历、医疗背景和工作经验？
3.其他相关信息的补充说明

二、慷慨大方的定义与经历
1.当您听到“慷慨大方”时，您会想到什么？下面这些词，是否会让您觉得与临床中的慷慨大方相关？
提供索引卡：大方、善意、良心、良医、同理心、人文关怀、道德义务、人情伦理、人道主义、社会责任、利他主义、某种赞誉\名声名望、宗教福报、日行一善、好人有好报等

2.您会花费额外的时间，或拿出自己的收入，来帮助那些有需要的患者吗？把这作为慷慨大方的定义，您觉得怎么样？

3.您认为自己可以在临床中展现这种慷慨大方吗？如果是，频次、程度怎么样？可以举个例子吗？例如经常、频繁地表达自己的慷慨大方，还是说很偶尔，要看情况，或者几乎不会这么做？

4.慷慨大方会对患者照料产生哪些积极或消极的影响? 对医疗系统的运作有哪些影响？
提示: 探索他们决定背后的原因 - 影响工作流程，不能解决每个人的问题，等等。

5.您周围有慷慨大方的同事吗？这对团队有什么影响？如果没有，提到“慷慨大方的临床医生”，有没有名声在外的榜样，或过去的人物、事件可以作为参考？
提示：对于个人——社会规范与同伴效应（感受到某种压力或期望，进而模仿这种行为）；对于团队——组织的凝聚力和效率得到了提升；一个良好的团队氛围；对于系统——更加合作、关爱和公平的医疗环境。

三、影响慷慨的因素
接下来，我们会讨论影响医护人员慷慨行为或沟通模式的潜在因素，包括医务工作者、患者和医疗体系三个层面。在临床经验方面，可能会提到患者的社会因素、患者行为、医患关系；在医护职业层面，可能涉及道德义务、职业自豪感和满足

感、职业倦怠；而在系统方面，可能有团队合作与支持、系统资源、薪水和其他因素（提供索引卡引入）。

1.我们将谈论临床经验中与慷慨大方相关的因素。
医生态度——
当患者向您倾诉他的难处时，您会做出什么口头或动作上的反应（例如口头安慰，点头表示理解，后续关注和随访）吗？为什么\为什么不这样做？
对于那些需求、请求超出了合理诊疗范围或医生职责的患者，您的态度和反应是什么？
医患互惠——
当您慷慨大方地对待患者时，他们会有什么反应或者回报呢？比如口头感谢，或行动上、情感上的反馈。
社会复杂性——
不同类型的患者会如何影响您的慷慨程度？比如他们的病种/病症、社会复杂性。社会复杂性包括特定或特殊群体、工作和生活环境等。探究：如果患者处于不同年龄段，或对方是女性、男男性行为者，性工作者等，会有什么不同。
污名化——
请展开谈谈社会复杂性，比如说，相比于一般的皮肤病患者，性病患者由于其面临的污名化和社会歧视，他们可能具有什么样的行为特征，以及这如何影响您作为临床工作者的慷慨行为？

2.接着，咱们来谈谈作为临床工作者，与慷慨大方有关的因素。
问责制——您是否觉得自己对患者预后负有责任？
感同身受——
您是否觉得自己关心着所有患者的健康、福祉？
您觉得怎样可以构建一个更加融洽的医患关系？
工作感受——
您觉得现在这份工作怎么样？您喜欢哪些方面？
不喜欢哪些方面？有哪些希望改进或改变的地方吗（从现实和理想层面考虑）？
这些因素是否促使您在工作中减少慷慨大方的行为？
职业倦怠/人格解体
如身心俱疲；消极怠工；注意力不集中；身体不适的各种症状；情感上的麻木、疲劳，甚至痛苦；不想听或不想关注任何事情，拒绝与他人的互动或共情。
您是否会感觉到职业倦怠呢？如果有，频率如何？
(·从不 ·一个月一次 ·一周一次 ·一周一次以上)
有什么情况可能导致您更容易倦怠呢？比如超负荷的工作量，患者各方面的复杂性、棘手程度或其他？
在倦怠时，会如何影响您对待患者或同事的慷慨？

3.系统层面上，哪些因素可能会影响到临床中的慷慨大方呢？
提供以下索引卡，让受访者选择对他们来说最相关的因素：
团队规模——
参与患者治疗的专业人员数量
人手配置是否能满足患者需求

资源——设备和技术是否足以满足患者需要
所有权——公立或私立医院
薪酬——工资高于或低于(省?)平均水平（方便的话，可以用跟我们说下您的年薪吗？）

4.什么因素会阻碍或促使您选择留在某个单位？
对年轻医护：您现在习惯了这份工作和内容吗？
对从业时间更长的医护:科研要求/行政要求/科务/与临床工作无关的文书（写报告）等，会如何影响您的工作状态和看法？
提供索引卡，让受访者选择对他们来说最相关的因素：
- 充足的支付和补偿（工资和奖金）
- 专业发展机会（教育、培训、职业前景）
- 工作保障（临时职位与永久职位/“编制”或铁饭碗）
- 工作环境（工作量、人员配备和支持）
- 工作条件和资源（必要的设备和高质量的设施）
- 行政要求/科务/与临床工作无关的文书等
- 位置（交通便利）
- 提供商、机构/单位的声誉（品牌和排名）

5.在您看来，上述提到的因素中，哪三种是最影响您在临床中对患者的慷慨大方的程度、行为的？
根据前面访谈提到的因素，提供索引卡，让受访者选择：
临床经验层面
个人职业层面
中观系统层面

6.您认为还有其他可能的因素，会影响临床中的慷慨大方吗？

7.我们可以采取哪些干预措施，去促进临床中更多的慷慨大方行为，减少和预防那些不良后果？

8.慷慨大方的行为可以怎样持续传递下去，影响他人呢？

结语：您还有其他问题或相关建议吗？
