# Supplementary material for: Situated generosity in clinical care: A mixed-methods study of STI services in China
Source: PLoS One. 2026 Jun 26;21(6):e0352469. doi: 10.1371/journal.pone.0352469 (PMC13308865; doi:10.1371/journal.pone.0352469)
Supplement: S3 File — (PDF) [file pone.0352469.s007.pdf]

S3 File. Thematic coding framework and csQCA calibration rules.

Project Title: Situated generosity in clinical care: A mixed-methods study of STI services in China

Methodology: Inductive Thematic Analysis & csQCA Calibration

Validation Strategy: Independent coding followed by negotiated consensus (100% agreement).

Part 1: Thematic Analysis Coding Scheme

This section details the inductive codes used to identify patterns in how participants conceptualize and experience generosity.

Code / Theme	Definition	Inclusion Criteria (When to apply)	Exclusion Criteria (When NOT to apply)	Representative Example (from Data)
1. Enacting Generosity				
1.1 Beyond Duty	Acts of care that explicitly exceed formal job descriptions, clinical protocols, or standard time allocations.	Code when participant mentions specific extra actions: giving money, skipping meals to see patients, extending clinic hours, or detailed stitching to minimize scars.	Do not code for standard good practice (e.g., prescribing correct meds, general politeness) or duties required by the hospital.	"Regardless of the time or pressure... she carefully sutures each patient, minimizing electrical damage. To me, this is very admirable." [D03]
1.2 Emotional Resilience	Maintaining a generous demeanor and providing emotional support even when physically tired or stressed.	Code when participant describes suppressing negative emotions to remain kind, or continuing to care despite burnout.	Do not code if the participant simply describes being happy/energetic (without the element of overcoming stress).	"When you're too tired, your tone might not be as gentle, but you have to control it... I still rely on my sense of responsibility to help." [L02]
2. Facilitators				

Code / Theme	Definition	Inclusion Criteria (When to apply)	Exclusion Criteria (When NOT to apply)	Representative Example (from Data)
2.1 Team & Role Modeling	References to the influence of mentors, leaders, or a supportive team culture on one's behavior.	Code when behavior is attributed to imitation of a senior doctor, team atmosphere, or peer support.	Do not code for general individual motivation unrelated to the social environment.	"The beliefs they passed on to me... I think that's a generous transmission... our team's concept can definitely influence each other." [S04]
2.2 Patient Reciprocity	Positive feedback loops where patient trust or compliance motivates the practitioner.	Code when participant explicitly links patient gratitude, trust, or cooperation to their own willingness to give more.	Do not code for general descriptions of "good patients" without linking it to the provider's reaction/generosity.	"If a patient is willing to accept the doctor's advice... I will feel more gratified... [and] be more proactive in providing follow-up services." [D06]
3. Barriers & Tensions				
3.1 Emotional Exhaustion / Depletion	Expressions of physical or emotional exhaustion that limit the capacity to be generous.	Code for terms like "exhausted," "drained," "numb," "irritated," or "reduced detail" in consultations due to volume.	Do not code for general complaints about salary or administrative tasks unless they explicitly link to emotional depletion.	"By the time you get to the 30th patient... the treatment is clearly less detailed. That’s definitely a sign of burnout." [S03]
3.2 Navigating Stigma	Challenges in dealing with difficult, arrogant, or stigmatized patients that test generosity.	Code when participant differentiates treatment based on patient attitude (arrogance) or social background (education, STD stigma).	Do not code for purely medical difficulties (e.g., complex surgery) that lack social/behavioral friction.	"For some men who are arrogant... I’m not generous with them... But for some vulnerable groups, I’m more generous." [N04]

Part 2: csQCA Calibration Rules (Dichotomization)

This section defines the "Anchors" used to convert qualitative data into crisp sets (0 or 1) for the truth table analysis. These rules serve as the inclusion/exclusion criteria for set membership.

Condition	Concept Definition	Anchor for 0 (Absence)	Anchor for 1 (Presence)
C1: SOCIAL COMPLEXITY	Attitude towards complex/stigmatized patients.<br>Does the practitioner maintain generosity towards patients with social complexity?	Exclusion Rule (0): Code as 0 if the practitioner explicitly states they withhold generosity from difficult/arrogant patients or "give up" on them.<br>Example: "I just find them really annoying... no matter what you say, they won't listen, so I just give up." [N04]	Inclusion Rule (1): Code as 1 if the practitioner expresses empathy specifically for the patient's social struggle or maintains care despite stigma.<br>Example: "In his eyes, every patient is important... Even dealing with stigmatized conditions, he remains patient." [N07]
C2: TEAM SUPPORT	Perception of collegial environment.<br>Does the practitioner report working in a supportive environment?	Exclusion Rule (0): Code as 0 if the participant mentions working in silos, lack of recognition from leaders, or purely transactional team relations.<br>Example: "The leaders haven't seen our achievements yet, which is difficult." [D05]	Inclusion Rule (1): Code as 1 if the participant explicitly mentions "unity," "family-like atmosphere," or specific help/mentorship from colleagues.<br>Example: "Everyone in this department is very happy... no one wants to leave." [N06]
C3: EMOTIONAL EXHAUSTION	State of Exhaustion.<br>Does the practitioner report significant fatigue or depersonalization?	Exclusion Rule (0): Code as 0 if the participant denies being tired, attributes fatigue only to physical hours (restable), or claims high resilience.<br>Example: "Clinical work isn't tiring, honestly. At most, it just costs extra time." [N01]	Inclusion Rule (1): Code as 1 if the participant uses strong emotional terms (drained, fed up, overwhelmed) or describes reducing quality of care.<br>Example: "It feels overwhelming... You really try to get everything done today." [N03]
C4: PATIENT COMPLIANCE	Perception of Patient Interaction.<br>Is the interaction defined by cooperation?	Exclusion Rule (0): Code as 0 if the narrative focuses on arguments, repetition, distrust, or "vexatious" patients.<br>Example: "They explain once, twice... and still, the patient doesn't stop." [S02]	Inclusion Rule (1): Code as 1 if the narrative focuses on mutual understanding, gratitude, or "manageable" patients.<br>Example: "Most patients are manageable... When they acknowledge my efforts... it brings me joy." [N08]
